# Mondo family proteins in diabetes

**DOI:** 10.3389/fendo.2025.1745162

**Published:** 2026-01-15

**Authors:** Sandeep Kumar Barodia, Maria B. Grant, Marina S. Gorbatyuk

**Affiliations:** 1 Department of Biochemistry, Wake Forest University Medical School, Winston-Salem, NC, United States; 2 Department of Ophthalmology, University of Alabama at Birmingham, Birmingham, AL, United States

**Keywords:** ChREBP, diabetes, glucose metabolism, insulin resistance, MondoA

## Abstract

Carbohydrate-responsive element binding protein (ChREBP) and Max-like protein X (MLX) are key Mondo Family Proteins (MFPs) acting as transcription factors. They are known to couple intracellular sugar levels with carbohydrate and lipid metabolism by regulating glucose-responsive gene expression. MondoA regulates lipid metabolism and insulin signaling in addition to controlling glucose uptake, whereas ChREBP primarily regulates *de novo* lipogenesis and glycolysis. Differential gene expression data suggest that the expression of MondoA and ChREBP is cell type-specific, which is an important consideration when designing therapeutic strategies to control MFP-regulated transcriptional programs for different tissue-specific systems. In this review, we summarize recent advances made in the research field studying diabetes and its multi-organ complications, including those affecting the kidney, liver, heart, and retina. We also discuss recent advances in MFP-targeted therapies.

## 1 Introduction

Glucose metabolism plays a pivotal role in fine-tuning cellular energy demands by regulating ATP production through glycolysis and oxidative phosphorylation, while also supplying precursors for the synthesis of essential cellular components. Recent studies highlight the critical importance of transcriptional regulation in maintaining both cellular and systemic energy homeostasis. Beyond serving as a metabolic substrate, glucose acts as a signaling molecule that activates specific transcriptional programs, thereby orchestrating gene expression to align cellular metabolism with nutrient availability. Among the key transcriptional regulators, MondoA and ChREBP (Carbohydrate Response Element-Binding Protein) sense intracellular glucose levels and modulate the expression of genes essential for sustaining energy balance. These two paralogous transcription factors, collectively known as the MondoA Family Proteins (MFPs), play a central role in glucose sensing and transcriptional control of energy metabolism. They regulate genes involved in glucose uptake, glycolysis, lipid biosynthesis, inflammation, and insulin signaling, effectively integrating nutrient-derived signals with metabolic output.

MondoA was first identified in 2000 by the group led by Donald Ayer (1). Initially described as MLX-interacting protein (MLXIP), MondoA was named for its ability to form heterodimers with MLX, a basic helix-loop-helix leucine zipper (bHLH-Zip) transcription factor. The MondoA–MLX complex binds to carbohydrate response elements (ChoREs), which include E-box motifs (CACGTG), in the promoters of glucose-responsive genes. This interaction enables transcriptional regulation in response to intracellular glucose metabolites, particularly glucose-6-phosphate (G6P). In parallel, a novel transcription factor gene named (1) (Williams-Beuren Syndrome Chromosomal Region 14) was cloned and characterized (2). Initially referred to as WS-bHLH, WBSCR14 encodes a protein with a bHLH-Zip domain and a bipartite nuclear localization signal, suggesting its role as a transcription factor. Subsequent studies revealed that WBSCR14 also forms heterodimers with MLX and binds ChoREs in a glucose-dependent manner, particularly regulating lipogenic gene expression. This protein was later renamed MLXIPL (MLX-interacting protein-like) and became widely known as ChREBP. ChREBP is now recognized as a central transcriptional regulator of glucose metabolism, especially in the liver and adipose tissue, where it activates genes involved in glycolysis and lipogenesis in response to elevated glucose levels.

MondoA and ChREBP were subsequently classified as MFPs, transcriptional activators structurally analogous to MYC. They share a conserved N-terminal domain found in Drosophila melanogaster and Caenorhabditis elegans. Unlike MYC, which is constitutively nuclear and promotes cell proliferation, MondoA and ChREBP are localized to the cytoplasm and mitochondria under basal conditions. Their nuclear translocation is tightly regulated by glucose-derived metabolites, especially G6P, positioning them as metabolic sensors that integrate signals from glucose metabolism. Tissue-specific expression patterns further distinguish these paralogs. ChREBP is predominantly expressed in the liver, white and brown adipose tissues, with moderate levels in the kidney, skeletal muscle, and small intestine (3). In contrast, MondoA is most abundantly expressed in skeletal muscle (1), suggesting distinct yet complementary roles in metabolic regulation. Additionally, MondoA forms a complex with MLC that supports mitochondrial function in primary skeletal muscle cells and erythroblasts by upregulating [*LDH-A, HKII, and PFKFB3*] genes (4), further underscoring its role in organelle-level metabolic integration.

Over the past two decades since the discovery of MondoA and ChREBP, our understanding of the structure and function of MFPs has significantly advanced, particularly in the context of metabolic disorders such as diabetes. Emerging evidence now indicates that these transcription factors also play critical roles in the pathogenesis of diabetic complications, including diabetic nephropathy, cardiomyopathy, and retinopathy. Their ability to integrate nutrient-derived signals with transcriptional programs positions them as key regulators of cellular responses to hyperglycemia and metabolic stress. In this review, we will examine the role of MFPs in the progression of diabetic complications and highlight existing gaps in MFP-based translational research, with the aim of identifying novel therapeutic opportunities and guiding future investigations.

## 2 Structure and functions of Mondo family proteins

### 2.1 Structure of ChREBP and MondoA proteins

**Figure 1:**
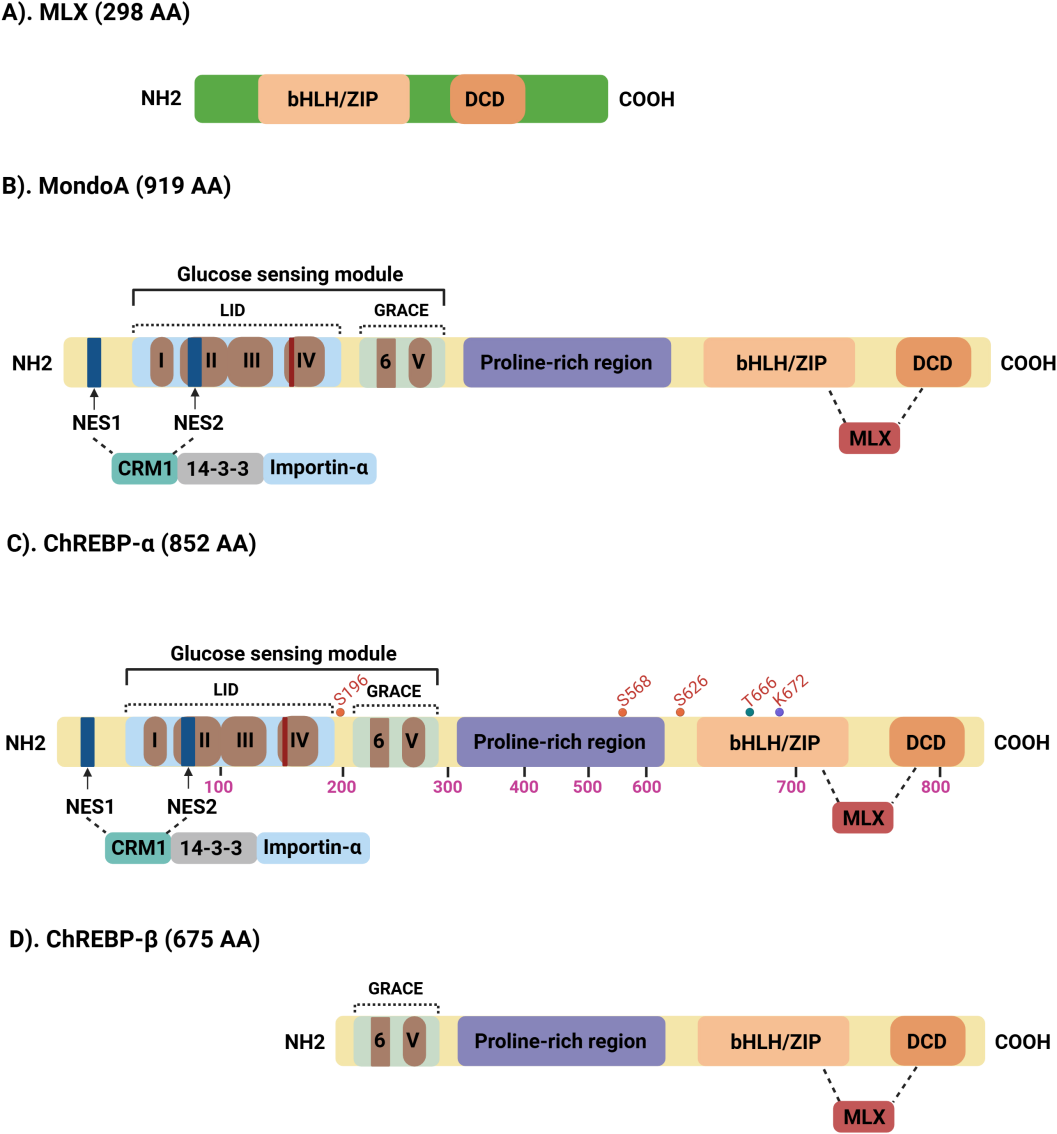
Schematic representation of structural domains of MFPs. **(A)** MLX (298 AA), MondoA and ChREBP (α or β isoforms) exhibit highly homologous C-terminal regions, including a basic helix-loop-helix/leucine zipper (bHLH/ZIP) domain and a cytoplasmic localization domain (DCD). The N-terminal regions of **(B)** MondoA (919 AA); **(C)** ChREBPα (852 AA) contain a glucose-sensing module (GSM), composed of five conserved Mondo regions (MCRI–V). This module is subdivided into a low-glucose inhibitory domain (LID) and a glucose-response activation conserved element (GRACE). Additionally, the N-terminal region includes (i) two CRM1-dependent nuclear export signals (NES1 and NES2), and (ii) a bipartite nuclear localization signal (NLS) that interacts with importin-α to mediate nuclear import. Between the GSM and the C-terminal domains, constitutive binding of MCRIII to 14–3-3 proteins contribute to cytoplasmic retention, transactivation, and nuclear export of MondoA and ChREBP. A variable proline-rich region is located centrally within the MondoA and ChREBPα sequences. **(D)**. ChREBPβ (675 AA) isoform, lacks the LID domain and MCRI-IV sequence.

Both MondoA and ChREBP are multi-domain proteins consisting of 919 and 852 amino acids, respectively. They share highly homologous N- and C-terminal regions, which include five Mondo conserved regions (MCR I–V), separated by a central proline-rich region (**Figure 1**). In their C-terminal domains, both proteins contain a basic helix–loop–helix–leucine zipper (bHLH/LZ) motif and a dimerization and cytoplasmic localization domain (DCD), which facilitate heterodimerization with MLX and enable DNA binding (5). Transcriptional activity by MondoA and ChREBP involves the formation of a heterotetrameric complex with MLX (6, 7).

The N-terminal domain primarily governs subcellular localization and transactivation in response to intracellular glucose levels. A key regulatory region within this domain is the glucose-sensing module (GSM), which is functionally conserved across both proteins. The GSM comprises two critical subdomains: the low-glucose inhibitory domain (LID) and the glucose-response activation conserved element (GRACE), spanning MCR I–IV and MCR V, respectively (8). The GRACE domain mediates transactivation, while the LID domain suppresses this activity under low-glucose conditions. Elevated glucose levels relieve this inhibition, enabling transcriptional activation. Notably, deletion of the LID domain results in constitutive activation of MondoA/ChREBP, independent of glucose availability. Mutational deletion of individual MCR I–IV abolishes MondoA/ChREBP transactivation in response to glucose, indicating that the entire MCR I–IV domain is essential for the glucose-responsive function of MFPs. The conserved spacing between MCR II, III, and IV further supports the structural integrity of this functional module. Repression of the GRACE domain by the LID domain is proposed to initiate an intramolecular interaction between MCR I–IV and MCR V, inducing a conformational change that inhibits DNA binding and transcriptional activation (9). Disruption of this repressive interaction permits direct binding of glucose metabolites G6P to the MCR I–IV region. Moreover, G6P has been shown to bind allosterically to Mondo proteins within the highly conserved MCR VI region, located within the GRACE domain. This region shares structural similarity with motifs found in glucose phosphate isomerase (GPI) and glutamine:fructose-6-phosphate aminotransferase (GFAT1), and due to its resemblance to a 9-amino-acid motif involved in CBP/p300 interaction, MCR VI has been designated as the transactivation domain (TAD) of Mondo proteins (10).

Detailed structural analyses of the N-terminal region of MondoA and ChREBP has revealed key regulatory elements: 1) Two CRM1-dependent nuclear export signals (NES1 and NES2), with NES2 localized within MCR II; 2) a bipartite nuclear localization signal (NLS) within MCR IV that binds importin-α to mediate nuclear import (11–13), and 3) constitutive binding of MCR III to 14–3–3 proteins, which contributes to cytoplasmic retention, transactivation regulation, and nuclear export of MondoA and ChREBP. Additionally, ChREBP is expressed as two isoforms ChREBP-α and ChREBP-β arising from alternative promoter usage and differing in their N-terminal regions. ChREBP-α contains the full regulatory architecture, including LID and GRACE domains, and is subject to glucose-dependent regulation. In contrast, ChREBP-β lacks the LID domain, resulting in constitutive transcriptional activity even under low-glucose conditions (**Figure 1**).

### 2.2 Physiological function of MondoA and ChREBP proteins

Both MondoA and ChREBP, play key regulatory roles in maintaining healthy physiological functions across multiple organs. Under healthy metabolic conditions, these MFPs are essential for maintaining glucose homeostasis. However, under chronic nutrient excess, their dysregulation contributes to the development of metabolic disorders, including obesity and diabetes. Both MFPs function in a coordinated manner to fine-tune the expression of regulatory genes involved in the vital metabolic functions. Thus, in response to elevated glucose flux, upon their nuclear translocation, both MFPs bind independently to the MLX protein and recognize ChoREs within the promoters of their target genes, thereby activating transcription. Among these target genes are those involved in lipid metabolism, such as acetyl-CoA carboxylase 1 (ACC), fatty acid synthase (FASN), and stearoyl-CoA desaturase-1 (SCD1), which collectively promote *de novo* lipogenesis. In addition to their overlapping roles in the regulation of lipid metabolism and TXNIP-mediated activation of nucleotide-binding domain, leucine-rich repeat, and pyrin domain-containing protein 3, mounting evidence suggests that they also play independent in a tissue-dependent context. Therefore, MFPs can be functionally distinguished by their tissue-dependent roles in metabolic pathway regulation. MondoA primarily drives lipid metabolism and insulin signaling, along with the regulation of TXNIP and ARRDC4-mediated glucose uptake (14, 15). In contrast, ChREBP primarily controls *de novo* lipogenesis and glycolysis (16, 17), although it is also known to regulate TXNIP transcription. For instance, high-fat diet in mice with liver-specific ChREBP overexpression leads to elevated serum triglycerides and inflammatory response (18, 19). Whereas a muscle-specific MondoA knockout, shows reduced expression of genes associated with insulin signaling and fatty acid metabolism (14). Moreover, under normal physiological conditions, MondoA–Mlx complex localizes to the outer mitochondrial membrane (OMM) (4). While ChREBP exhibits punctate cytoplasmic localization in hepatocytes under low glucose condition (20).

**Figure 2:**
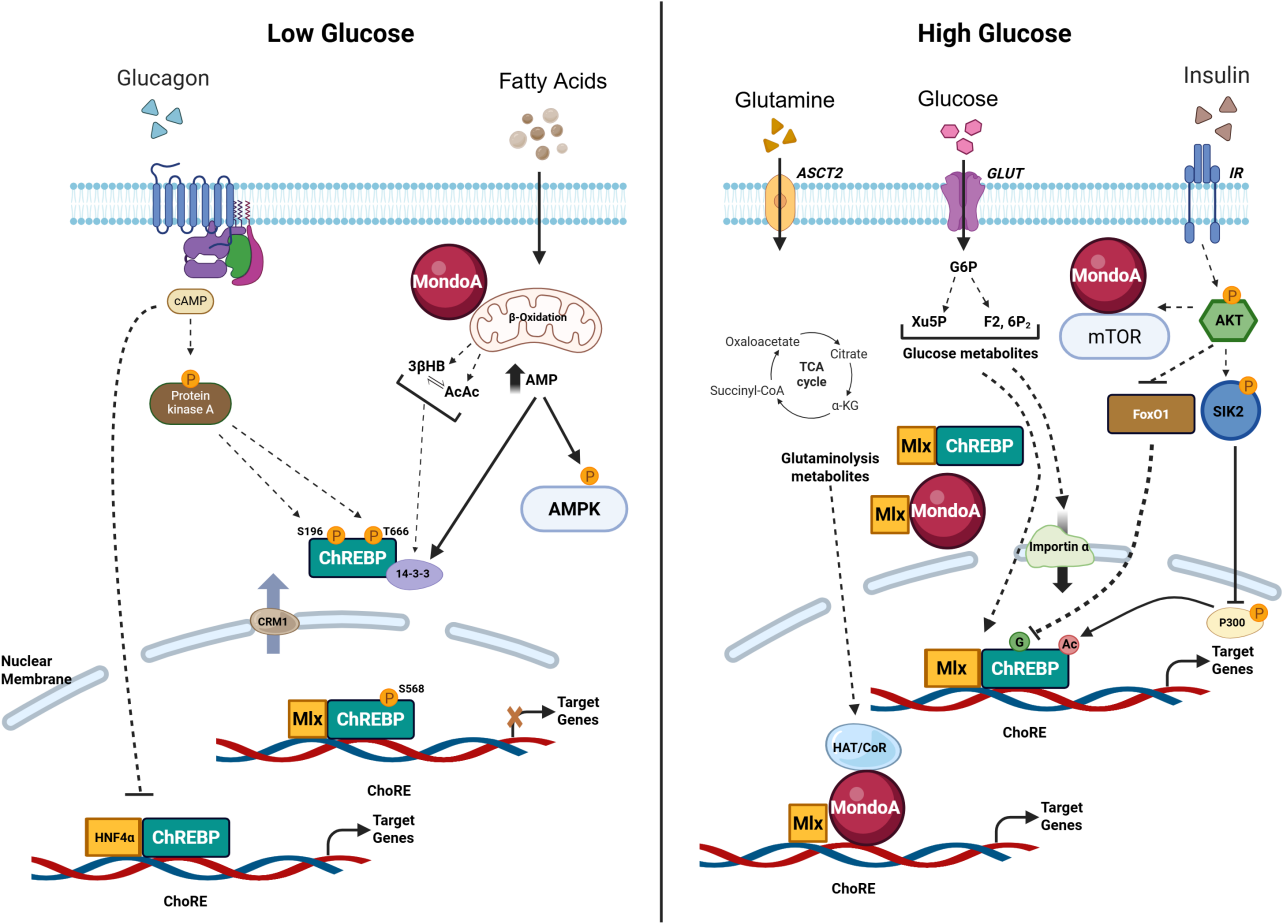
Physiological functions of MondoA/ChREBP under varying nutrient conditions. Activation of MFPs requires nuclear translocation, followed by activation of DNA binding by Mondo/MLX complexes. Mondo proteins are regulated by nuclear/cytosol trafficking mediated by interactions with 14–3–3 proteins, CRM-1, or importin-α. In response to low glucose concentrations, intracellular cAMP upon glucagon release activates PKA, which phosphorylates ChREBP on (Ser 196 and Thr 666) residues located in the NLS and the bHLH/LZ domains, thereby inhibiting ChREBP nuclear import and DNA binding activity, respectively. cAMP also acts by disrupting a ChREBP-HNF4α-CBP complex at target gene promoters in a β-cell line. MondoA has been reported to be localized to the outer mitochondrial membrane under low glucose conditions. In contrast, under high glucose condition, glucose is phosphorylated into glucose-6-phosphate (G6P), which enters glycolysis and the pentose phosphate pathway, generating xylulose-5-phosphate (Xu5P). ChREBP nuclear translocation and DNA binding is facilitated by PP2A-mediated dephosphorylation of Ser196 and Ser568/Thr666. Xu5P selectively activates PP2A in high-glucose environments. G6P may also act as an allosteric activator of ChREBP, while fructose-2,6-bisphosphate (F2,6P2) has emerged as a key metabolite in glucose-induced recruitment of ChREBP and MondoA to target genes. Additionally, glutamine—converted to glutamate and α-ketoglutarate in the TCA cycle has been shown to inhibit MondoA transcriptional activity. Insulin is known to enhance ChREBP activity by inhibiting FoxO1, which increases ChREBP O-GlcNAcylation and stability. Conversely, insulin can suppress MondoA activity via (i) activation of SIK2, which blocks ChREBP acetylation at Lys672 by p300, thereby promoting DNA binding, and (ii) mTOR binding to cytosolic MondoA, preventing MondoA/MLX dimerization.

MFPs follow a well-defined intracellular pathway in response to elevated glucose conditions, wherein both MondoA and ChREBP undergoes heterodimerization with Mlx, followed by nuclear translocation and binding to ChoREs (21–25). However, the activity of ChREBP and MondoA is tightly regulated at multiple levels as glucose-induced nuclear accumulation alone is insufficient for full transactivation of their target genes (21, 22). Several lines of evidence entail that ChREBP/MondoA–Mlx complex activation is triggered by G6P (**Figure 2**) (26, 27), which is generated after glucose is metabolized by hexokinases or glucokinase that are also localized to the OMM (28). Application of a glucose analog 2-deoxyglucose (2-DG) demonstrates the specific responsiveness of MondoA–Mlx complex to G6P. Notably, 2-DG is phosphorylated into 2-deoxyglucose-6-phosphate (2-DG6P), but it fails to be processed further, leading to 2-DG6P accumulation. While 2-DG itself is a glucose analog and not metabolized beyond phosphorylation, its conversion to 2-DG6P is sufficient to activate MondoA signaling. Upon exposure to 2-DG, the MondoA–Mlx complex translocate into the nucleus and potently transactivates target genes (23).

The role of xylulose 5-phosphate (Xu5P) in mediating ChREBP activation is controversial. Earlier studies that Xu5P activates the ChREBP–Mlx complex by stimulating protein phosphatase 2A (PP2A), which in turn promotes dephosphorylation and activation of the complex (29). However, other findings reported that PP2A activity is dispensable for ChREBP activation in response to glucose, and that dephosphorylation on Ser-196 is not sufficient to promote ChREBP nuclear translocation without increased glucose metabolism. This study reveals that G6P, rather than its downstream metabolite Xu5P, is essential for both ChREBP nuclear translocation and transcriptional activity in response to glucose in liver cells (27). Thus, activation by G6P appears to be a common feature of both MondoA–Mlx and ChREBP–Mlx complexes. As a distinguished feature, the MondoA–Mlx complex also responds to other phosphorylated hexoses, such as allose, 3-O-methylglucose and glucosamine (30), while ChREBP has been shown to be activated by fructose 2,6-bisphosphate in hepatocytes (31).

## 3 Mondo family proteins in obesity and diabetes

MFPs play crucial role in regulating nutrient metabolism and maintaining energy homeostasis. These transcription factors are central to nutrient sensing in key metabolic organs such as skeletal muscle, liver, adipose tissue, and pancreas. Under normal nutrient conditions, they help preserve metabolic balance. However, under nutrient overload, MondoA and ChREBP directly regulate glucose and lipid metabolism in brown and beige adipocytes and modulate inter-organ communication to coordinate systemic metabolic responses. Dysregulation of these pathways contributes to the development of metabolic diseases, including obesity, insulin resistance, and type 2 diabetes mellitus (T2DM) (32). Their activity in thermogenic adipose tissues is particularly important for counteracting obesity and related disorders (3, 33). For example, MondoA’s regulatory function is enhanced under high glucose conditions, which paradoxically impairs insulin sensitivity across various tissues. In skeletal muscle, elevated glucose and fructose levels activate MondoA, leading to the transcription of genes that inhibit insulin signaling and glucose uptake (34). Notably, mice lacking MondoA in skeletal muscle exhibit improved insulin sensitivity and enhanced glucose uptake (35). ChREBP, on the other hand, exhibits tissue-specific and metabolic context-dependent functions. Chronic activation of ChREBP can result in “glucolipotoxicity”, a condition marked by cytotoxicity due to excessive glucose and lipid accumulation. In the liver, high glucose levels activate ChREBP, which induces genes involved in glycolysis and *de novo* lipogenesis, contributing to hepatic steatosis as observed in non-alcoholic fatty liver disease (NAFLD). In adipose tissue, ChREBP promotes triglyceride storage, and while its overexpression can protect against obesity and insulin resistance, dysregulation may provoke inflammation. Interestingly, unlike in rodents, ChREBP is not the primary glucose sensor in human pancreatic β-cells. However, chronic overexpression of the hyperactive isoform ChREBPβ in mouse models has been linked to β-cell apoptosis, loss of β-cell identity, and diabetes (36).

### 3.1 Mondo family proteins in diabetic kidney disease

MFPs play a complex role in diabetic kidney disease (DKD), primarily by regulating glucose and lipid metabolism within kidney cells (**Table 1**). Dysregulation of both ChREBP and MondoA under diabetic conditions can lead to cellular damage and metabolic imbalance, driving the progression of kidney disease. Several reports suggest that ChREBP plays important roles in the onset and progression of diabetic nephropathy (37–39)., which is well known to develop after long-term duration suffering from diabetes mellitus. ChREBP contributes significantly to DKD pathogenesis due to its overactivation in high-glucose environments. Studies have shown that ChREBP and its pro-inflammatory target TXNIP are overexpressed in the kidneys of both human patients and animal models of T2D. Another study demonstrated that ChREBP-β and TXNIP exacerbate fructose-induced renal injury by triggering ferroptosis in renal tubular epithelial cells (40). By promoting TXNIP expression, the dysregulated ChREBP pathway induces oxidative stress and apoptosis in kidney cells, ultimately leading to diabetic nephropathy. Genetic knockout of ChREBP in mice has been shown to improve kidney function, reduce cell death, and decrease oxidative stress and inflammation in a diabetic environment (28, 41)

**Table 1:** Studies investigating the role of MFPs in diabetes.

Disease model	MondoA/ ChREBP	Mechanism	Pathology	Reference
Obesity- induced T2D	ChREBP	ChREBP-β overexpression downregulated expression of genes involved in mitochondrial biogenesis, autophagy, and respiration.	Thermogenic gene expression (e.g. Dio2, UCP1) is markedly inhibited in brown adipose tissue overexpressing ChREBP-β.	Wei et al., 2020 (33)
Diabetic Kidney	ChREBP	ChREBP-β mediates fructose-induced ferroptosis of renal tubular epithelial cells.	ChREBP-β overexpression leads to ferroptosis of renal tubular epithelial cells and kidney injury.	Guo et al., 2023 (40)
Diabetic Liver	ChREBP	Lipogenic gene expression is strongly associated to the expression of ChREBP-β and to metabolic risk markers in both white adipose tissue and liver.	Lipogenic enzymes and the GLUT-4 are markedly decreased in white adipose tissue of insulin-resistant obese individuals.	Eissing et al., 2013 (42)
Diabetic Heart	MondoA	Nuclear translocation of MondoA upregulates *Arrdc4* transcriptional expression, increased lysosomal GLUT1 trafficking, and blocked glucose transport in cardiomyocytes, thus forming a feedback mechanism.	ARRDC4 regulates hyperglycemia-induced toxicities toward cardiac and skeletal muscle	Nakayama et al., 2024
Diabetic Retina	ChREBP MondoA and ChREBP	High glucose is associated with elevated production of HIF-1α-induced VEGF. MondoA is prominently expressed in cones, while ChREBP is broadly expressed throughout the retina.	Retinal neovascularization Elevated expression of both proteins was observed in DR.	Chang et al., 2014; Starr et al., 2025. (46, 47)

### 3.2 Mondo family proteins in diabetic liver disease

MFPs play a crucial role in diabetic liver disease, with ChREBP emerging as the dominant regulator driving the metabolic dysregulation that leads to NAFLDa common consequence of diabetes and insulin resistance (**Table 1**). Under hyperglycemic conditions, MFPs activate the transcription of key enzymes involved in *de novo* lipogenesis, resulting in increased triglyceride synthesis and hepatic lipid accumulation, ultimately causing hepatic steatosis or fatty liver (42). This condition is now more accurately referred to as metabolic dysfunction-associated steatotic liver disease (MAFLD). Hepatic ChREBP activation contributes to a vicious cycle of worsening insulin resistance. In diabetic mouse models, liver-specific inhibition of ChREBP improves hepatic insulin sensitivity, whereas its overexpression induces steatosis (13). Notably, the ChREBP isoform β is markedly upregulated in diabetic livers and further enhances lipid synthesis, exacerbating insulin resistance. The liver is particularly susceptible to fructose-induced lipogenesis, a process strongly controlled by ChREBP. High-fructose diets significantly elevate ChREBP activity and are recognized as major contributors to NAFLD progression (43). Although MondoA is predominantly expressed in skeletal muscle, it also plays an important role in diabetic liver disease through inter-organ metabolic crosstalk, functioning in concert with ChREBP. Activated MondoA promotes lipid synthesis and ectopic lipid accumulation, which substantially contribute to systemic hyperglycemia and hyperlipidemia key conditions that further damage the liver. Preclinical studies show that inhibiting MondoA with compounds like SBI-477 can diminish triglyceride levels and alleviate hepatic steatosis, thus highlighting the significance of muscle-liver communication in diabetic liver disease (15). The prominent and distinct roles of ChREBP and MondoA in mediating metabolic dysregulation enlightens them as appealing therapeutic targets for treating diabetes and associated liver disease. Considering its direct involvement in hepatic lipogenesis, inhibiting ChREBP activity in the liver could reduce fat accumulation and improve insulin sensitivity. Furthermore, selective inhibition of MondoA activity could restore systemic insulin resistance and lower the burden of hyperglycemia and hyperlipidemia on the liver. Additional research studies are required to explore the complex interactions and context-dependent effects of MFPs to develop targeted therapeutic strategies.

### 3.3 Mondo family proteins in coronary heart disease

Dysregulation in the MFPs activity has been implicated in heart diseases, particularly those associated with metabolic disorders. Studies in Drosophila have explored the connection between altered nutrient metabolism and cardiac hypertrophy using genetic tools. Notably, manipulation of molecular pathways involved in glucose and lipid metabolism led to hypertrophic growth in fly hearts (44). The conserved nature of the Mondo-Mlx network and its downstream targets suggests a similar role in human cardiac hypertrophy. Given its high expression in cardiac and skeletal muscle tissues, MondoA is the principal MFP influencing cardiac function. It has been shown to be essential for normal myogenesis and regulation of skeletal muscle glycogen content in mice (34). Cardiac physiology relies heavily on the ability to switch between glucose and fatty acids for energy production. In many cardiac disorders, this dynamic is impaired, a condition known as metabolic inflexibility. The MondoA-Mlx complex is a key regulator of this dynamic, modulating gene expression in response to changes in glucose availability (4). Under hyperglycemic conditions, activation of the MondoA-Mlx complex promotes the conversion of excess glucose into lipids, which can lead to ectopic triglyceride accumulation in non-adipose tissues, including the heart contributing to cardiac dysfunction and insulin resistance. Moreover, metabolic diseases such as T2DM and obesity are major risk factors for cardiovascular diseases. Thus, MondoA-Mlx signaling pathway represents a promising therapeutic target for metabolic and cardiac complications. In mouse models, small molecule inhibitors of MondoA (SBI-477/993) have demonstrated improved glucose tolerance, enhanced insulin sensitivity, and increased glucose uptake in skeletal muscle—highlighting their potential to prevent metabolic dysfunctions that contribute to heart disease (15). However, further research is needed to elucidate the tissue-specific roles and mechanisms of MondoA-Mlx signaling in cardiac pathology. Tissue-specific knockout models and gene-editing approaches may pave the way for targeted therapies for distinct forms of heart disease (**Table 1**).

### 3.4 Mondo family proteins in diabetic retinopathy

Due to their key regulatory function in diabetes, MFPs could be explored to better understand the underlying mechanisms of diabetic retinopathy (DR). Although earlier studies have extensively explored the roles of both MFPs in several human metabolic disorders, only a few relevant research reports have focused on the role of ChREBP in the vision field, and to the best our knowledge, no studies to date have investigated MondoA—thus indicating a gap in our understanding of these proteins in the retina (**Table 1**) (45, 46). It has been proposed that ChREBP-mediated and normoxic HIF-1α activation may be partially responsible for neovascularization in diabetic and age-related retinopathy (46). Furthermore, ChREBP deficiency has been shown to reduce high-glucose-induced apoptosis, migration, and tube formation in human retinal microvascular endothelial cells as well as structural and angiogenic responses in the mouse retina (45). We have demonstrated for the first time that overexpression of MFPs is linked to the progression of DR (47). In this recent study, we showed that ChREBP and MondoA are expressed throughout the retina and upregulated in humans patients and in murine model of DR. Under hyperglycemic conditions, elevated levels of both MondoA and ChREBP in the retina lead to cellular metabolic reprogramming, which contributes to photoreceptor deterioration and vision loss. Mice with constitutively active ChREBP (caChREBP) in photoreceptors exhibit significant changes in retinal protein expression, affecting metabolic, structural, and signaling pathways, further supporting the role of ChREBP in DR. Furthermore, MFPs also regulate genes involved in inflammatory pathways, and their increased activity in the hyperglycemic environment of DR may contribute to the inflammatory processes during disease progression. These findings suggest that targeting ChREBP and MondoA could be a promising strategy for developing novel treatments for DR. Further research is warranted to fully understand which specific retinal cell types and ocular conditions would benefit most from targeting MFPs.

## 4 Targeting Mondo family proteins in diabetes

MFPs are emerging as promising therapeutic targets for diabetes. Current preclinical strategies focus on inhibiting MondoA in skeletal muscle and modulating ChREBP’s activity in the liver and adipose tissue to address insulin resistance in a tissue-specific manner. Notably, ChREBP can also initiate beneficial signaling pathways, such as the induction of fibroblast growth factor 21 (FGF21), which enhances insulin sensitivity (48). Given ChREBP’s dual physiological roles, therapeutic approaches aim to modulate rather than completely inhibit its activity. Liver-specific ChREBP inhibitors have shown promise in reducing hepatic fat accumulation and improving insulin sensitivity (13). However, because ChREBP also contributes to protective metabolic function, a well-tuned strategy is required to avoid adverse effects. For instance, selectively modulating ChREBP signaling could preserve its beneficial pathways while limiting its lipogenic activity. Additionally, targeting the ChREBP–FGF21 axis in diabetes could influence insulin sensitivity and blood glucose levels, which are already dysregulated under diabetic conditions (49). Therefore, future MFP-based therapies must carefully weigh the potential benefits against the risks of modulating MFP activity across various diabetic organs and tissues.

In white adipose tissue, ChREBP regulates the synthesis of insulin-sensitizing lipids such as palmitic-acid-hydroxy-stearic-acids (PAHSAs). In obese and diabetic individuals, reduced adipose ChREBP expression and PAHSA production contribute to systemic insulin resistance (50). Restoring ChREBP function in adipose tissue may therefore enhance systemic insulin sensitivity. Therapeutic approaches should emphasize activating adipose ChREBP or enhancing PAHSA biosynthesis to improve metabolic outcomes. In pancreatic β cells, MFPs play a complex, context-dependent role. While MondoA is essential for glucose-stimulated gene expression, prolonged CHREBP activation can lead to β-cell dysfunction and apoptosis—a phenomenon known as glucolipotoxicity. One potential strategy involves the selective inhibition of MondoA to reduce TXNIP-mediated β-cell apoptosis. A more refined approach to modulating ChREBP in β-cells may involve balancing the expression of its isoforms, ChREBP-α and ChREBP-β, to promote cell proliferation without triggering apoptosis. Developing compounds that can target MFPs in a tissue-specific manner such as inhibiting MondoA in muscle while activating ChREBP in adipose tissue remains a major challenge in precision drug development. The lack of resolved crystal structures for key domains of MondoA and ChREBP limits the design of potent and selective modulators. Applications of SBI-477 or its recent analog SBI-993, which exhibits enhanced potency and improved pharmacokinetic properties, along with inhibitors of ChoRE-driven transcriptional activity, could significantly advance research on the role of ChREBP and MondoA in diabetes (51). For example, leveraging this approach may help address critical questions regarding the therapeutic potential of MFP-based strategies in DR.

Furthermore, due to the diverse tissue-specific expression and function of these proteins, systemic administration of therapeutic agents often could often lead to undesirable side effects. Future research should focus on characterizing the full metabolic profile of MFPs across tissues, which will be critical for the rational design of targeted therapies. Advances in tissue-specific knockout models and gene-editing technologies may also pave the way for precision interventions targeting diabetic complications across multiple organs.
